# Efficacy and Functional Outcomes of Platysma Myocutaneous Flap in Oral and Maxillofacial Reconstruction

**DOI:** 10.7759/cureus.70464

**Published:** 2024-09-29

**Authors:** Abhijeet Humne, Rashmi Agarwal, Ashish Uppal, Rohan Mehra, Hemant Gupta, Hemant Mehra

**Affiliations:** 1 Department of Oral and Maxillofacial Surgery, Babu Banarasi Das College of Dental Sciences, Lucknow, IND; 2 Department of Electrical Engineering and Computer Science, Indian Institute of Science Education and Research, Bhopal, IND

**Keywords:** efficacy, functional outcomes, oral cancer, platysma, reconstruction

## Abstract

Introduction: Maxillofacial reconstruction strives to restore the form and function of the maxillofacial region in patients with deformities caused by trauma, disease, cancer, or congenital malformations. An array of surgical procedures has been advised for restoring soft tissues in the oral cavity, with varied degrees of efficacy. This prospective study seeks to provide a thorough understanding of the success rate, complications, functional outcomes, and patient satisfaction associated with the utilization of platysma myocutaneous flap (PMF) in this specialized domain.

Aim: This study aims to elaborate on the PMF surgical technique, along with the flap design, outcomes, and intricacies associated with age, gender, the recipient site, and defect size.

Materials and methods: The study included 30 patients with oral malignancies who intervened under general anesthesia with wide local excision and reconstruction with PMF at a single tertiary care center.

Results: The results showed that the PMF is an efficient flap in the context of its ease of harvest, minimal donor site morbidity, low complication rates (infection - 26.67%, flap dehiscence - 16.67%, and flap necrosis - 6.77%), and ability to rehabilitate both form and function.

Conclusion: In conclusion, the PMF stands as a viable alternative in oral and maxillofacial reconstruction, offering a combination of versatility, reliable blood supply, and potential for functional restoration.

## Introduction

Background

Oral and maxillofacial reconstruction involves the recuperation of form and function in patients with deformities caused by trauma, cancer resection, congenital anomalies, or other pathological conditions. An efficient repair of soft tissue discrepancies in the oral and maxillofacial region is crucial for the restoration of facial aesthetics, oral function, and overall quality of life. A multitude of surgical approaches have been developed to address these complex reconstructions, including the use of local, regional, and distant flaps [1]. One such flap is the platysma myocutaneous flap (PMF), which utilizes the platysma muscle and overlying skin to reconstruct soft tissue defects in this region.

The history of the PMF dates back to 1887, when an Austrian surgeon Robert Gersuny [2] documented reconstructing a full-thickness cheek deformity using a cervical skin/platysma flap that was turned inside out to create a new buccal mucosa lining. This was perhaps the earliest description of the PMF used for head and neck reconstruction. However, it was not until 1978 that the PMF was put forward by Futrell et al. as an appealing reconstructive approach with various advantages [3]. Despite providing acceptable outcomes with an acceptable degree of morbidity, this flap has been less frequently utilized as a result of the increasing use of microvascular free tissue transfer. Experience gained by surgeons over the years has demonstrated that microvascular flaps are not always suitable and that, in some circumstances, the PMF not only provides an excellent reconstructive result but also serves as a viable alternative to microvascular flaps.

The principal constraints of the PMF are its dearth of thickness and its partial dependence on the facial artery. According to Persky et al. [4], this flap was traditionally avoided if a prior neck dissection had been done or if a ligation of the facial artery was required. Nevertheless, McGuirt et al. were the pioneers in disputing this and proving a noteworthy collateral flow [5].

The PMF is easy to harvest, thin, and versatile, allowing for three-dimensional reconstruction with a low donor site morbidity following primary neck closure [6,7]. It is large enough to close most ablative lesions in the head and neck up to 70 cm^2^, and there is no additional special equipment required. Total or partial necrosis of the skin island, fistula, dehiscence, hematoma, and cellulitis are among the possible complications when using this technique, with the complication percentage varying from 18% to 45% [8].

There are many alternatives available for reconstructing oral cavity surgical defects. The variables that affect the choice of a reconstructive procedure include the size of the defect, donor site morbidity, functional outcome, and concerns about aesthetics. The PMF offers several advantages, including its proximity, robust blood supply, and versatility in addressing various defect types. However, a comprehensive evaluation of the efficacy and functional outcomes of the PMF in oral and maxillofacial reconstruction is still warranted. This study will serve as an essential resource for oral and maxillofacial surgeons, providing a deep understanding of the advantages and challenges associated with the utilization of PMFs in reconstructive procedures.

Research objectives

This research aimed to provide a comprehensive understanding of the success rate, complications, functional outcomes, and patient satisfaction associated with the utilization of PMFs in this specialized field.

Research questions

To accomplish the research objectives, the following research questions will determine the investigation: (1) What is the success rate of the PMF in oral and maxillofacial reconstruction? (2) What are the complications associated with the utilization of the PMF in oral and maxillofacial reconstruction? (3) What are the functional outcomes, including oral function and aesthetic results, of the PMF in oral and maxillofacial reconstruction? (4) What is the level of patient satisfaction and quality of life following the utilization of the PMF in oral and maxillofacial reconstruction?

Significance of the study

In the field of oral and maxillofacial reconstruction, this study holds a significant importance both clinically and academically. It comprehensively evaluates the efficacy and functional outcomes of the PMF. Additionally, this study will serve as a useful resource for subsequent research, encouraging further investigations into the utilization of the PMF and enhancing the overall knowledge within the domain of oral and maxillofacial reconstruction.

## Materials and methods

Methodology

Thirty cases of PMF were used to restore post-cancer resection deformities in the oral and maxillofacial regions. The analysis included all information about the functional and aesthetic results, tumor locations, defect types, donor and recipient sites, complications at the recipient site, and surgical management of these patients undergoing PMF reconstruction. All patients were treated at a single institution. We utilized the "University of Washington - Quality of Life (UW-QOL) 1999" version 4 questionnaire to assess the functional efficacy results.

Materials

Cases with stage I-III diseases involving hard and soft tissue of the maxillofacial region that require composite resection and reconstruction with a pedicled flap were selected. All the patients were informed about the nature of the surgical and experimental procedures, and consent was obtained before surgery.

Inclusion criteria

Patients who consented to participate in the study and were newly diagnosed with squamous cell carcinoma cases classified as stage I-III according to the American Joint Committee on Cancer (AJCC) Eighth Edition TNM (tumor/node/metastasis) Classification (2017), with a maximum staging of T3 N1 M0, and who had not undergone previous surgery and radiotherapy, were included. Additionally, patients with lesions necessitating soft tissue reconstruction were also eligible for inclusion in the study.

Exclusion criteria

Patients unwilling to participate in the study, those for whom surgery was contraindicated, those who require special health care, or those with recurrent cases of malignancy were excluded from the study.

Statistical analysis

The data for the present study was entered in Microsoft Excel 2007 (Microsoft Corp., Redmond, WA) and analyzed using the SPSS statistical software v23.0 (IBM Corp., Armonk, NY). The descriptive statistics included frequency and percentage. The level of significance for the present study was fixed at 5%. Chi-square tests and paired t-tests were used to ascertain if the variables were associated.

## Results

This study selected representative cases through a retrospective review of surgical records, focusing on patients who demonstrated similar indications for PMF. The selection process considered factors such as defect size, location, and patient demographics. This methodology ensured that the findings reflect a broad spectrum of clinical scenarios.

Case 1

**Figure 1:**
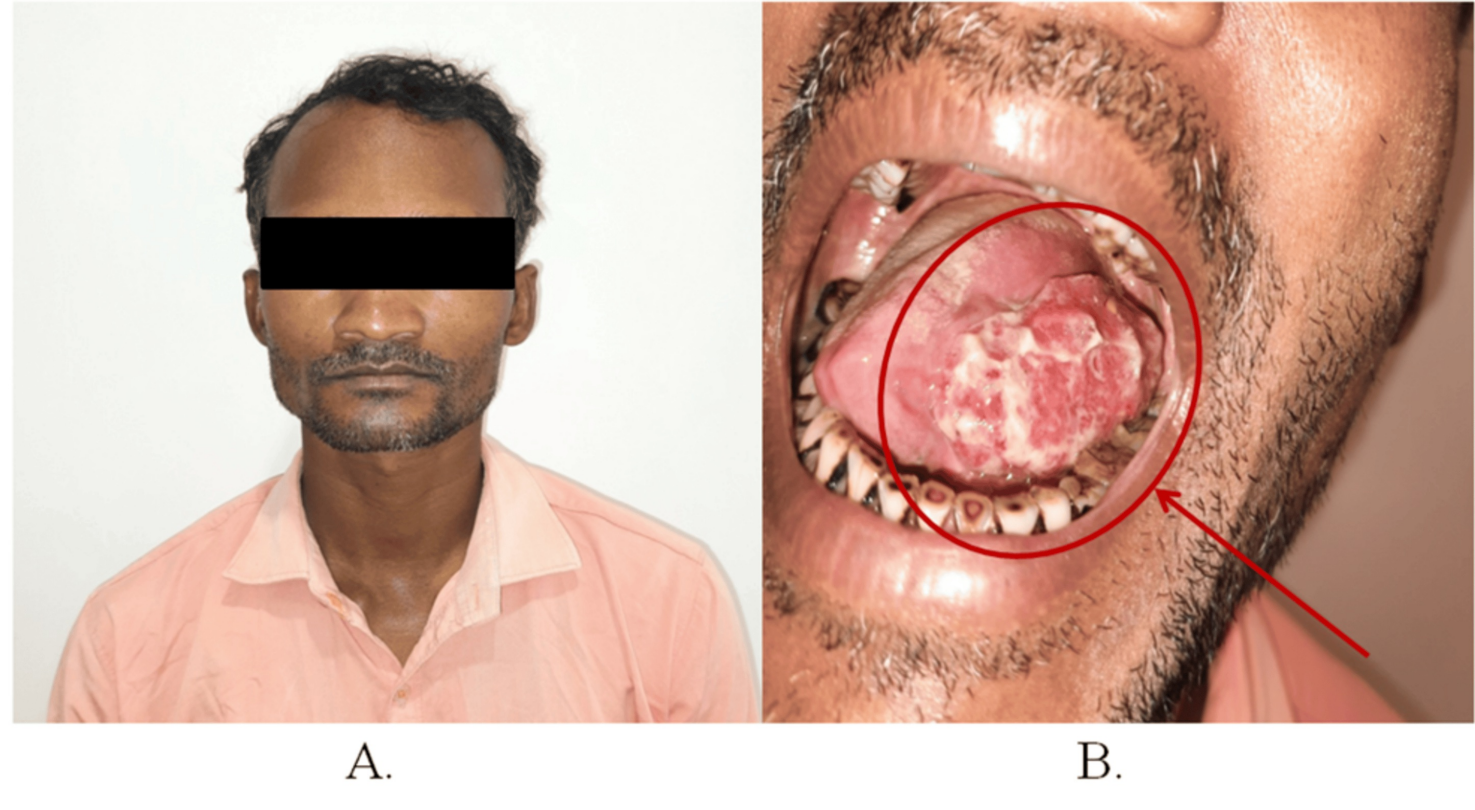
Preoperative photographs of patient 1 (A) Frontal profile and (B) intraoral profile.

A 38-year-old male patient visited the department with the chief complaint of growth and pain in the left lateral border of the tongue region for two to three years. Clinical evaluation revealed an ulcero-proliferative growth on the left lateral tongue, with lymph nodes palpable up to level IB (Figure 1, Panels A and B).

**Figure 2:**
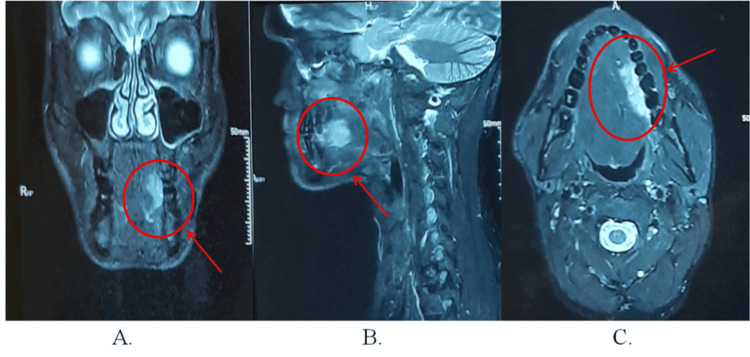
Preoperative radiographs of patient 1 (A) Coronal section of CE-MRI scan (face), (B) sagittal section of CE-MRI scan (face), and (C) axial section of CE-MRI scan (face).

Contrast-enhanced magnetic resonance imaging (CE-MRI) showed a well-defined, heterogeneously enhancing soft tissue lesion involving the anterior two-thirds of the left lateral border of the tongue, with concurrent cervical lymphadenopathy (Figure 2, Panels A-C).

**Figure 3:**
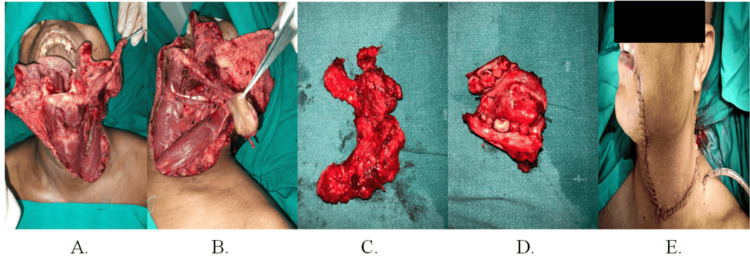
Intraoperative photographs of patient 1 (A) Recipient site surgical defect, (B) exposure of skin paddle of PMF, (C) resected lymph nodes, (D) resected tumor, and (E) reconstruction of the recipient site defect with PMF. PMF: Platysma myocutaneous flap.

The patient underwent wide local excision (WLE) with marginal mandibulectomy and removal of lymph nodes up to level III. A pedicled PMF was utilized, with careful preservation of the branches of the external jugular vein (EJV) (Figure 3, Panels A-E).

**Figure 4:**
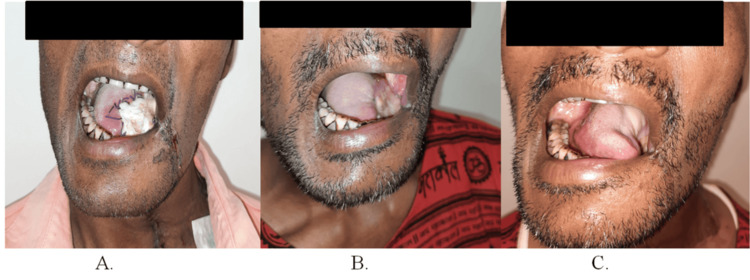
Postoperative photographs of patient 1 (A) One-week intraoral profile, (B) one-month intraoral profile, and (C) six-month intraoral profile.

Layer-wise closure of the defect was done using PMF through an appropriately sized tunnel. Postoperative follow-ups at one week, one month, and six months confirmed adequate mucosalization of the flap (Figure 4, Panels A-C).

Case 2

**Figure 5:**
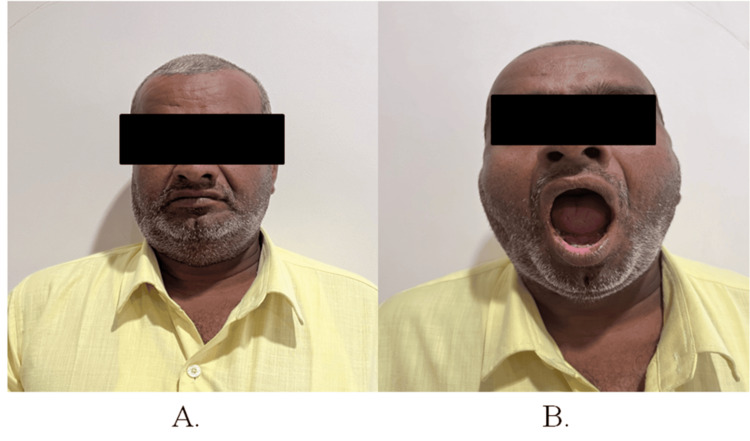
Preoperative photographs of patient 2 (A) Frontal profile and (B) intraoral profile.

A 47-year-old male patient presented to the department with a chief complaint of pain and sensitivity to spicy and hot foods in the right buccal region for the past one to two years. Clinical examination revealed an ulcero-proliferative growth on the right buccal mucosa with palpable lymphadenopathy extending to level IB (Figure 5, Panels A and B).

**Figure 6:**
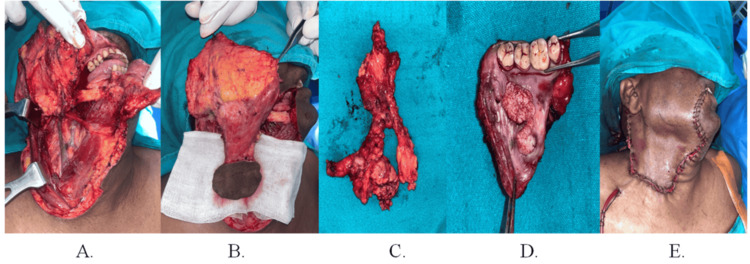
Intraoperative photographs of patient 2 (A) Recipient site surgical defect, (B) exposure of skin paddle of PMF, (C) resected lymph nodes, (D) resected tumor, and (E) reconstruction of the recipient site defect with PMF. PMF: Platysma myocutaneous flap.

The patient underwent WLE along with marginal mandibulectomy and removal of lymph nodes up to level III. A pedicled PMF was harvested with careful preservation of the branches of the facial nerve and EJV. The surgical defect was closed with layer-by-layer approximation of the PMF through an appropriately sized tunnel (Figure 6, Panels A-E).

**Figure 7:**
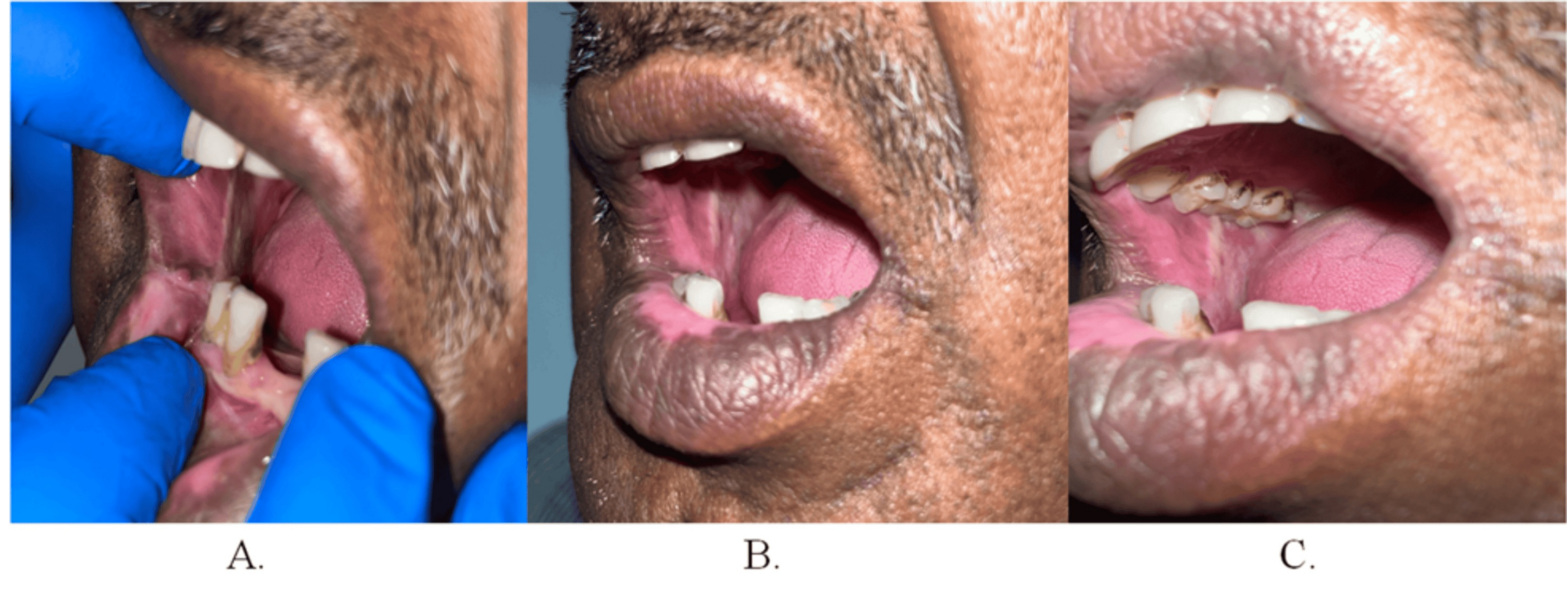
Postoperative photographs of patient 2 (A) One-week intraoral profile, (B) one-month intraoral profile, and (C) three-month intraoral profile.

Postoperative flap evaluation was conducted at one week, one month, and three months, with satisfactory mucosalization observed at each interval (Figure 7, Panels A-C).

Infection

At the one-week time interval, 26.67% of the subjects developed infections. However, at one month, three months, and six months, none of the subjects had infections, which was statistically significant. There was no discernible correlation between age or gender and flap viability (P < 0.001), as shown in Table 1.

**Table 1:** Intragroup comparison of infection between different time intervals

	Absent	Present	Chi-square value	P-value	Significance
1 week	22	08	25.714	0.021	Significant
	73.33%	26.67%			
1 month	30	0			
	100.0%	.0%			
3 months	30	0			
	100.0%	.0%			
6 months	30	0			
	100.0%	.0%			

Flap dehiscence

At the one-week time interval, 16.67% of the subjects experienced flap dehiscence, while none of the subjects had flap dehiscence at one month, three months, and six months. The difference between the one-week and one-month intervals, one-week and three-month intervals, and one-week and six-month intervals was statistically significant (P < 0.001), as shown in Table 2.

**Table 2:** Intragroup comparison of flap dehiscence between different time intervals

	Absent	Present	Chi-square value	P-value	Significance
1 week	25	05	15.653	0.001	Significant
	83.33%	16.67%			
1 month	30	0			
	100.0%	.0%			
3 months	30	0			
	100.0%	.0%			
6 months	30	0			
	100.0%	.0%			

Flap necrosis

At the one-week time interval, 6.77% of the subjects experienced flap necrosis, while none of the subjects had flap necrosis at one month, three months, and six months, which was non-significant (P > 0.05), as shown in Table 3. The patients experienced a partial necrosis (50%) of the epithelium alone. The underlying platysma muscle remained viable, and with bedside debridement done after two to three weeks, these flaps healed by secondary intention.

**Table 3:** Intragroup comparison of flap necrosis between different time intervals

	Absent	Present	Chi-square value	P-value	Significance
1 week	28	02	6.102	0.106	Non-significant
	93.33%	6.77%			
1 month	30	0			
	100.0%	.0%			
3 months	30	0			
	100.0%	.0%			
6 months	30	0			
	100.0%	.0%			

Pain

At the one-week time interval, 93.3% of the subjects experienced moderate pain that required regular medication. At the one-month interval, 53.3% had no pain and 46.7% had mild pain. There was a statistically significant reduction in the pain from one week to one month and three months (P < 0.001), as shown in Table 4.

**Table 4:** Intragroup comparison of pain between different time intervals

	Score 1	Score 2	Score 3	Score 4	Score 5	Chi-square value	P-value
1 week	0	2	28	0	0	141.200	0.001 (Significant)
	.0%	6.7%	93.3%	.0%	.0%		
1 month	16	14	0	0	0		
	53.3%	46.7%	.0%	.0%	.0%		
3 months	29	1	0	0	0		
	96.7%	3.3%	.0%	.0%	.0%		
6 months	29	1	0	0	0		
	96.7%	3.3%	.0%	.0%	.0%		

Shoulder activity

About 20% of the patients had stiff shoulders in the first week, but that did not affect their activity or strength. At one and three months, 13.3% and 6.7% had stiff shoulders, respectively; however, at six months, none of the subjects had problems with the shoulder (P < 0.001), as shown in Table 5.

**Table 5:** Intragroup comparison of shoulder between different time intervals

	Score 1	Score 2	Score 3	Score 4	Chi-square value	P-value
1 week	24	6	0	0	8.713	0.001 (Significant)
	80.0%	20.0%	.0%	.0%		
1 month	26	4	0	0		
	86.7%	13.3%	.0%	.0%		
3 months	28	2	0	0		
	93.3%	6.7%	.0%	.0%		
6 months	10	0	0	0		
	100.0%	.0%	.0%	.0%		

Mood

At the one-week time interval, 26.7% were somewhat depressed about cancer, and 23.3% were neither in a good mood nor depressed about cancer. By one month, 76.7% of patients had a good mood, and by three and six months, 90% had a generally good mood and were occasionally depressed by cancer. There was a statistically significant improvement in mood from one week to one month to three months (P < 0.001). From three to six months, the mood was consistently good, and there was no statistically significant difference (Table 6).

**Table 6:** Intragroup comparison of mood between different time intervals

	Score 1	Score 2	Score 3	Score 4	Score 5	Chi-square value	P-value
1 week	0	15	7	8	0	37.225	0.001 (Significant)
	.0%	50.0%	23.3%	26.7%	.0%		
1 month	0	23	7	0	0		
	.0%	76.7%	23.3%	.0%	.0%		
3 months	0	27	03	0	0		
	.0%	90.0%	10.0%	.0%	.0%		
6 months	0	27	03	0	0		
	.0%	90.0%	10.0%	.0%	.0%		

Daily activities

At the one-week and one-month time intervals, 80% of the subjects reported difficulty in activity, stating they were unable to keep their old pace but not very often. However, at three and six months, 96.7% of subjects had no difficulty in their daily activities. There was a significant improvement in the activity from one month to three and six months. Between one week and one month, there was no significant change in the activity (P = 0.002), as shown in Table 7.

**Table 7:** Intragroup comparison of activity between different time intervals

	Score 1	Score 2	Score 3	Score 4	Score 5	Chi-square value	P-value
1 week	0	24	6	0	0	14.773	0.002 (Significant)
	.0%	80.0%	20.0%	.0%	.0%		
1 month	0	23	7	0	0		
	.0%	76.7%	23.3%	.0%	.0%		
3 months	0	29	1	0	0		
	.0%	96.7%	3.3%	.0%	.0%		
6 months	1	29	0	0	0		
	3.3%	96.7%	.0%	.0%	.0%		

Chewing

At one week, 90% of the subjects were unable to chew even soft solids. At one month, 63.3% were unable to chew soft solids, and 36.7% were able to chew soft solids. At three and six months, 63.3% and 73.3% were able to chew soft solids. There was a statistically significant improvement in chewing from one week to one month to three to six months (P < 0.001), as shown in Table 8.

**Table 8:** Intragroup comparison of chewing between different time intervals

	Score 1	Score 2	Score 3	Chi-square value	P-value
1 week	0	3	27	69.126	0.001 (Significant)
	.0%	10.0%	90.0%		
1 month	0	19	11		
	.0%	63.3%	36.7%		
3 months	7	19	4		
	23.3%	63.3%	13.3%		
6 months	8	22	0		
	26.7%	73.3%	.0%		

## Discussion

The intricate architecture and functional significance of the involved structures in following resection render reconstruction of the residual defects extremely challenging in the field of oral and maxillofacial surgery. For patients undergoing reconstructive procedures, numerous surgical approaches have been implemented to restore form and function. The use of PMF is one such method that is gaining attention as a reconstructive option.

While previous authors only documented random cervical platysma flaps, Futrell et al. were the first to describe a true musculocutaneous cervical flap that preserved the mandibular insertions, which was composed of a lower cervical skin paddle and platysma muscle [3,5]. PMF has been used to reconstruct a wide range of head and neck defects. It has been proven to be an effective surgical alternative for reconstructing small- to medium-sized (15-75 cm^2^) lesions of the oral and maxillofacial region and large-sized defects in rare cases [9,10]. The thin and flexible characteristics of the muscle-skin paddle unit render the platysma flap highly appropriate for customizing repairs of defects in the floor of the mouth, cheek mucosa, and gum. This adaptability helps mitigate postoperative functional challenges stemming from primary closure under excessive tension [11,12].

This study adds to the existing literature supporting the role of the PMF in the reconstruction of defects in the maxillofacial region. In our experience of 30 patients, the PMF was extremely reliable with minimal complications. Overall, the quality of life in these patients was very good. The mean operating time of less than four hours for tumor resection and PMF reconstruction underscores the efficiency of using this flap. The exact time necessary for the flap elevation was not documented, but it does not add materially to the time required for neck dissection itself. The facial artery and/or its branches were preserved when possible [13-15]. It should be highlighted that, in our study, all 30 patients had either a segmental or marginal mandibulectomy performed as part of the tumor excision.

Although patients undergoing other reconstructive procedures, like the pectoralis major flap or the radial forearm free flap, were not formally evaluated in this study, we are confident that the PMF engenders less donor site morbidity than these two more commonly utilized flaps. Therefore, we advocate for greater consideration and use of the PMF for reconstructing maxillofacial defects.

The PMF offers several advantages and is generally adequate for most soft tissue resections, except for extensive cases like total glossectomy. The flap is thin and flexible, allowing it to conform well to the defect. Its arc of rotation enables it to reach beyond the midline in the pharynx and slightly past the midline of the anterior floor of the mouth. It can be combined with other reconstructive techniques [16]. Harvesting the flap takes only about 15 minutes more than the standard elevation of subplatysmal neck flaps. Closing the donor site is straightforward, involving the undermining of the superior chest wall skin over the clavicle. Even if there is a partial loss of skin at the distal donor site, the final appearance of the neck remains unchanged after neck dissection, and no functional issues are related to the neck. The drawbacks of platysma flaps are relatively minimal.

Preservation of facial vessels, if deemed necessary, may restrict the use of PMF in cases of bulky neck node metastases [17]. While the study did not directly investigate this concern, some literature suggests that facial vessels may not be essential for flap survival. Nevertheless, due to the precarious blood supply to the skin paddle, there is a notable rate of postoperative complications [16]. Complications such as infections, flap dehiscence, flap necroses, fistula formation, and skin paddle slough were observed during our study. These complications were closely monitored and documented to ensure a thorough evaluation of patient outcomes. In this study, however, these complications did not negatively impact the treatment cost or the ultimate achievement of reconstructive objectives.

Complications such as infection, fistula formation, and skin paddle slough were effectively addressed through conservative management, relying solely on supportive care. In the immediate postoperative period, if the skin paddle appears white, it often indicates an impending skin slough. Recent studies have reported skin slough incidence rates as high as 60%. Consistent with earlier research, our study also recorded a 40% incidence of skin slough, which was managed conservatively. Typically, when sloughing occurs, the underlying muscle remains viable. In cases where the flap is used intraorally, skin sloughing can facilitate mucosalization and lead to a more natural long-term outcome, devoid of hair growth in the mouth and excessive contraction [18,19]. However, skin sloughing may have more pronounced aesthetic consequences when the flap is utilized for facial reconstruction.

To prevent hematoma formation at the donor site, employing a suction drain is recommended. The drain should remain in position until the output diminishes to less than 30 ml within a 24-hour time frame [8]. During this time, the patient refrains from oral intake until the closure of the flap is guaranteed.

Flap dehiscence was seen in five cases at week 1, mostly in small defects (5-6 cm in diameter). Local wound care or secondary closure was the treatment of choice, when this complication occurred, with an acceptable outcome. Notably, no additional secondary surgical interventions were needed for any of the cases.

Several parameters were utilized to evaluate the functional outcomes of the patients based on the University of Washington questionnaires. In our study, at one week, one month, three months, and six months, 70% of individuals had difficulty pronouncing certain words, but their speech remained understandable over the phone, while 30% experienced speech difficulty to the extent that only family and friends could understand them.

At one week, 90% of the subjects were not able to chew even soft solids. At one month, 63.3% were not able to chew soft solids, and 36.7% were able to chew soft solids. At three and six months, 63.3% and 73.3% were able to chew soft solids. Moreover, at six months, 73.33% could swallow without difficulty. Additionally, 76.7% of the patients were able to taste the majority of the food items normally.

At the one-week time interval, 93.3% of the subjects had moderate pain that required regular medication. At one month, 53.3% had no pain, and 46.7% had mild pain. At three and six months, 96.7% of the subjects experienced pain. There was a statistically significant reduction in the pain from one week to one month and three months. At one week, one month, three months, and six months, 20%, 13.3%, 6.7%, and 0% of the patients, respectively, experienced a stiff shoulder; however, this did not affect their activity or strength.

Moreover, at the one-week time interval, 40% of the patients were somewhat apprehensive about cancer, while 30% were neither optimistic nor despairing about cancer. At one month, three months, and six months, 70% had generally good mood and were occasionally depressed by cancer. A statistically significant improvement was observed in the patient’s mood from one week to one month. From one month to three months and six months, the mood was consistently good, and no statistically significant difference was seen.

To enhance our insights into complications and their correlation with various factors, a larger sample size would have been essential for this study. For precise guidelines on reconstructing different parts of the oral cavity, comparisons with other reconstruction methods are essential. Consequently, the occurrences of postoperative complications and their effectiveness are considerably lower than what is required for firm conclusions. Nonetheless, despite the limitations in sample size, we endorse an increased consideration and utilization of the PMF for reconstructing defects in the oral cavity.

## Conclusions

In conclusion, the PMF is a valuable option in oral and maxillofacial reconstruction, offering a combination of versatility, reliable blood supply, and potential flap for functional restoration. As surgical techniques continue to evolve, the PMF stands as a testament to the progress in achieving optimal outcomes in the challenging field of oral and maxillofacial surgery. A comprehensive evaluation of functional outcomes and comparisons with alternative techniques contribute to a holistic understanding of the role of PMF in the evolving landscape of oral and maxillofacial reconstruction. Future research should focus on refining techniques, minimizing complications, and exploring synergies with emerging technologies to further enhance the efficacy of this reconstructive approach.

## Appendices

This study incorporated the University of Washington Quality of Life (UW-QOL) 1999" version 4 questionnaire to assess the efficiency of the flap in our study.

University of Washington Quality of Life Questionnaire (UW-QOL)

This questionnaire asks about your health and quality of life over the past seven days. Please answer all of the questions by checking one box for each question.

1. Pain (Check one box)

i. I have no pain.

ii. There is mild pain - do not need medication.

iii. I have moderate pain - that requires regular medication (codeine or non-narcotic).

iv. I have severe pain controlled only by narcotics.

v. I have severe pain, not controlled by medication.

2. Appearance (Check one box)

i. There is no change in my appearance.

ii. The change in my appearance is minor.

iii. My appearance bothers me but I remain active.

iv. I feel significantly disfigured and limit my activities due to my appearance.

v. I cannot be with people due to my appearance.

3. Activity (Check one box)

i. I am as active as I have ever been.

ii. There are times when I can't keep up my old pace, but not often.

iii. I am often tired and have slowed down my activities although I still get out.

iv. I don't go out because I don't have the strength.

v. I am usually in bed or chair and don't leave home.

4. Recreation (Check one box)

i. There are no limitations to recreation at home or away from home.

ii. There are a few things I can't do but I still get out and enjoy life.

iii. There are many times when I wish I could get out more, but I'm not up to it.

iv. There are severe limitations to what I can do, mostly I stay at home and watch TV - I can't do anything enjoyable.

5. Swallowing (Check one box)

i. I can swallow as well as ever.

ii. I cannot swallow certain solid foods - I can only swallow liquid food.

iii. I cannot swallow because it "goes down the wrong way" and chokes me.

6. Chewing (Check one box)

i. I can chew as well as ever.

ii. I can eat soft solids but cannot chew some foods.

iii. I cannot even chew soft solids.

7. Speech (Check one box)

i. My speech is the same as always.

ii. I have difficulty saying some words but I can be understood over the phone.

iii. Only my family and friends can understand me.

iv. I cannot be understood.

8. Shoulder (Check one box)

i. I have no problem with my shoulder.

ii. My shoulder is stiff, but it has not affected my activity or strength.

iii. Pain or weakness in my shoulder has caused me to change my work.

iv. I cannot work due to problems with my shoulder.

9. Taste (Check one box)

i. I can taste food normally.

ii. I can taste most foods normally.

iii. I can taste some foods.

iv. I cannot taste any foods.

10. Saliva (Check one box)

i. My saliva is of normal consistency.

ii. I have less saliva than normal, but it is enough.

iii. I have too little saliva.

iv. I have no saliva.

11. Mood (Check one box)

i. My mood is excellent and unaffected by my cancer.

ii. My mood is generally good and only occasionally affected by my cancer.

iii. I am neither in a good mood nor depressed about my cancer.

iv. I am somewhat depressed about my cancer.

v. I am extremely depressed about my cancer.

12. Anxiety (Check one box)

i. I am not anxious about my cancer.

ii. I am a little anxious about my cancer.

iii. I am anxious about my cancer.

iv. I am very anxious about my cancer.

Which issues have been the most important to you during the past seven days? Check up to three boxes.

~ Pain ~ Swallowing ~ Taste

~ Appearance ~ Chewing ~ Saliva

~ Activity ~ Speech ~ Mood

~ Recreation ~ Shoulder ~ Anxiety

General questions

1. Compared to the month before you developed cancer, how would you rate your health-related quality of life? (Check one box)

~ Much better

~ Somewhat better

~ About the same

~ Somewhat worse

~ Much worse

2. In general, would you say your health-related quality of life during the past seven days has been: (Check one box)

~ Outstanding

~ Very good

~ Good

~ Fair

~ Poor

~ Very poor

3. Overall quality of life includes not only physical and mental health, but also many other factors, such as family, friends, spirituality, or personal leisure activities that are important to your enjoyment of life. Considering everything in your life that contributes to your personal well-being, rate your overall quality of life during the past seven days. (Check one box)

~ Outstanding

~ Very good

~ Good

~ Fair

~ Poor

~ Very poor

Please describe any other issues (medical or nonmedical) that are important to your quality of life and have not been adequately addressed by our questions (you may attach additional sheets if needed).
